# Sequential Au(I)-catalyzed reaction of water with *o*-acetylenyl-substituted phenyldiazoacetates

**DOI:** 10.3762/bjoc.7.74

**Published:** 2011-05-18

**Authors:** Lei Zhou, Yizhou Liu, Yan Zhang, Jianbo Wang

**Affiliations:** 1 Beijing National Laboratory of Molecular Sciences (BNLMS), Key Laboratory of Bioorganic Chemistry and Molecular Engineering of Ministry of Education, College of Chemistry, Peking University, Beijing 100871, China

**Keywords:** alkyne, carbene O–H insertion, cyclization, diazo compounds, gold catalysis, 1*H*-isochromene

## Abstract

The gold(I)-catalyzed reaction of water with *o*-acetylenyl-substituted phenyldiazoacetates provides 1*H*-isochromene derivatives in good yields. The reaction follows a catalytic sequence of gold carbene formation/water O–H insertion/alcohol-alkyne cyclization. The gold(I) complex is the only catalyst in each of these steps.

## Introduction

Transition metal carbene complexes are versatile intermediates and can undergo diverse transformations, including X–H (X = C, O, S, N, etc.) insertions, cyclopropanations, ylide formation, and 1,2-migrations [1–5]. Among the various methods to generate metal carbene complexes, transition metal-catalyzed decomposition of diazo compounds is the most straightforward and is highly reliable. Various transition metals have been found to decompose diazo compounds and then transfer a carbene unit to saturated or unsaturated organic substrates [3]. However, compared to the other group 11 metals, i.e., copper and silver, there are only a few reports on gold-catalyzed carbene transfer reactions of diazo compounds [6–20]. In 2005, Díaz-Requejo, Nolan, Pérez and co-workers reported the first example of carbene transfer from ethyl diazoacetate (EDA) using (IPr)AuCl. The subsequent generation of a gold carbene was followed by insertion into a phenyl C–H bond, an O–H bond, or an N–H bond [6]. Similar reactions were also reported by Dias and co-workers with a gold(I) ethylene complex [7]. Although the scope of those studies was limited to ethyl diazoacetate, the examples therein demonstrated that gold complexes can be used as efficient catalysts in carbene transfer reactions with diazo compounds.

**Scheme 1:**
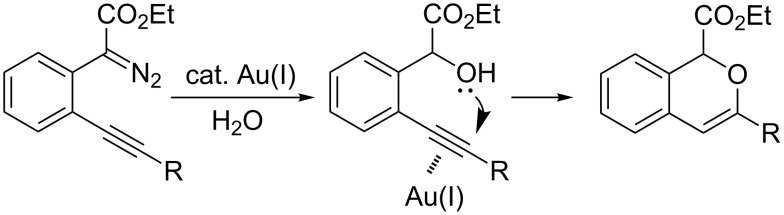
Gold(I)-catalyzed insertion/cyclization of *o*-acetylenyl-substituted phenyldiazoacetates providing 1*H*-isochromene derivatives.

On the other hand, the development of reaction systems in which a single catalyst mediates two or more different reactions in a selective manner has become an emerging area of research [21–28]. This type of sequential or concurrent catalysis is particularly appealing in view of the requirements of green chemical processes in the fine chemical industry. In this context, we have previously reported the copper(I)-catalyzed reaction of amines with *o*-acetylenyl-substituted phenyldiazoacetates, which leads to a Cu(I)-catalyzed tandem N–H insertion/hydroamination of an alkyne [29]. Subsequently, we have tried to extend this reaction by replacing the amine component with water, and we expected that similar tandem reaction would occur. Copper is a good catalyst for the decomposition of diazo compounds and the subsequent insertion of water. However, we have found that it is not a suitable catalyst for the alcohol–alkyne cyclization. Since gold complexes are well-known for their efficacy in activating alkynes, we reasoned that a concurrent catalysis based on gold-catalyzed reaction of diazo compounds and alkynes might be possible [30]. Herein we report such a catalytic system, namely a gold(I)-catalyzed insertion/cyclization cascade by reacting water with *o*-acetylenyl-substituted phenyldiazoacetates. The reaction affords 1*H*-isochromene derivatives in good yields (Scheme 1) [31].

## Results and Discussion

At the onset of this investigation, *o*-acetylenyl-substituted phenyldiazoacetate **1a** was selected as the model substrate. In a preliminary experiment, **1a** was treated with CuI catalyst in a mixture of CH_3_CN and H_2_O (v:v = 1:1) (Table 1). As expected, only the water insertion product **4a** was obtained as the major product and in high yield (91%) (Table 1, entry 1). Since previous reports have shown that silver [32–34] and gold [35–38] complexes are efficient catalysts for alcohol–alkyne cyclization, we then proceeded to examine other catalysts viz. AgOTf, AgF/PCy_3_, NaAuCl_4_ and AuCl (Table 1, entries 2–5). However, these metal complexes were not efficient catalysts for the decomposition of diazo compounds, and a large amount of starting materials remained. However, when **1a** was treated with (PPh_3_)AuCl in a mixture of CH_3_CN and H_2_O, product **4a** was obtained in 80% yield, along with the minor cyclization product **2a** (8%) (Table 1, entry 6). Encouraged by this result, electron-rich ligand coordinated gold complexes (PMe_3_)AuCl and (IPr)AuCl were examined (Table 1, entries 7 and 8). It was found that (IPr)AuCl was an efficient catalyst for both carbene transfer and cyclization. Product **2a** from 6-*endo-dig* cyclization and product **3a** from 5-*exo-dig* cyclization were both obtained in nearly equal amounts in combined yield of 95%. Next, we examined the effect of the ratio of CH_3_CN and H_2_O with (IPr)AuCl as catalyst: A ratio of 1:1 (v:v) afforded the best results (Table 1, entries 9–12). It is worth noting that the reaction also occurred in pure water to afford the cyclization products, **2a** and **3a**, in moderate yield (Table 1, entry 11). Next, the effect of different co-solvents, such as DMF, NMP (*N*-methylpyrrolidone) and toluene, was investigated (Table 1, entries 13–15). Interestingly, when a mixture of H_2_O and DMF (v:v = 1:1) was used as the solvent, the ratio of **2a**:**3a** increased to 4:1, with slightly diminished overall yield. The reaction with NMP or toluene as the co-solvent gave poor yields of desired products (Table 1, entries 14,15). Finally, the effect of temperature was evaluated: The reaction gave diminished yields when carried out at a temperature higher or lower than 80 °C (Table 1, entries 16–18).

**Table 1:** Optimization of reaction conditions with phenyldiazoacetate **1a**^a^.

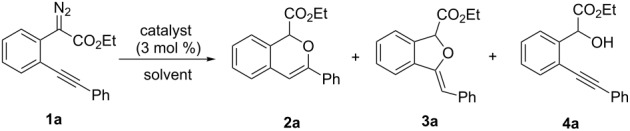						

Entry	Catalyst	Solvent	*T*/°C	Yield (**2a** + **3a**)^b^	**2a**:**3a**	**4a**, Yield

1	CuI	H_2_O:CH_3_CN (1:1)	80	0%	—	91%
2	AgOTf	H_2_O:CH_3_CN (1:1)	80	0%	—	<10%
3	AgF/PCy_3_	H_2_O:CH_3_CN (1:1)	80	trace	—	trace
4	NaAuCl_4_	H_2_O:CH_3_CN (1:1)	80	0%	—	trace
5	AuCl	H_2_O:CH_3_CN (1:1)	80	0%	—	trace
6	(PPh_3_)AuCl	H_2_O:CH_3_CN (1:1)	80	8%	—	80%
7	(PMe_3_)AuCl	H_2_O:CH_3_CN (1:1)	80	10%	—	15%
8	(IPr)AuCl	H_2_O:CH_3_CN (1:1)	80	95%	1:1	0%
9	(IPr)AuCl	H_2_O:CH_3_CN (1:3)	80	25%	2:3	61%
10	(IPr)AuCl	H_2_O:CH_3_CN (3:1)	80	57%	1:1.3	11%
11	(IPr)AuCl	H_2_O	80	54%	1:2	0%
12^c^	(IPr)AuCl	CH_3_CN	80	0%	0	41%
13	(IPr)AuCl	H_2_O:DMF (1:1)	80	90%	4:1	0%
14	(IPr)AuCl	H_2_O:NMP (1:1)	80	0%	—	trace
15	(IPr)AuCl	H_2_O:toluene (1:1)	80	19%	1:1	0%
16	(IPr)AuCl	H_2_O:DMF (1:1)	100	57%	3:1	0%
17	(IPr)AuCl	H_2_O:DMF (1:1)	60	66%	5:1	0%
18	(IPr)AuCl	H_2_O:DMF (1:1)	40	48%	5:1	0%

^a^All the reactions were carried out using 0.2 mmol phenyldiazoacetate **1a** with 3 mol % of catalyst in 1 mL solvent for 24 h. ^b^Yield and ratio of **2a** and **3a** were measured by ^1^H NMR. ^c^1 mmol of water was added.

With the optimized reaction conditions in hand, a series of substituted diazo compounds **1a**–**f** were prepared, and their reactions with water in the presence of (IPr)AuCl in aqueous DMF were investigated. As shown in Table 2, all the reactions gave isochromene derivatives as the major products. In the reaction of diazo compounds **1e** and **1f** with water, only 6-*endo-dig* cyclization products **2e** and **2f** were isolated as the sole product in yields of 69% and 75%, respectively. Functional groups such as bromo and hydroxy groups were tolerated under the present catalytic systems. When diazo compound **1g** (R’ = H) was employed as the substrate, none of the cyclization product was detected and the water insertion product **4g** was obtained in 81% yield.

**Table 2:** Gold(I)-catalyzed cascade insertion/cyclization of water with various substituted phenyldiazoacetates^a^.

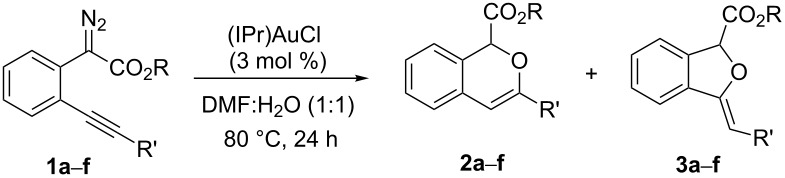			

Entry	Substrate	Yield of **2**^b^	Yield of **3**^b^

1	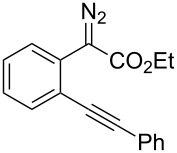 **1a**	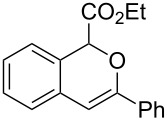 **2a**, 70%	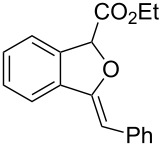 **3a**, 15%
2	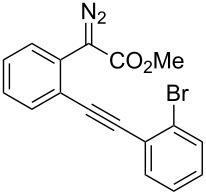 **1b**	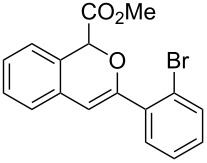 **2b**, 64%	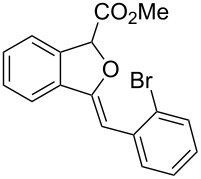 **3b**, 31%
3	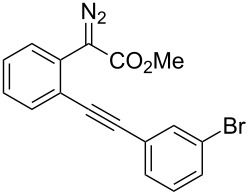 **1c**	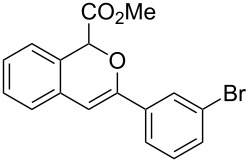 **2c**, 67%	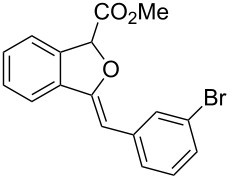 **3c**, 22%
4	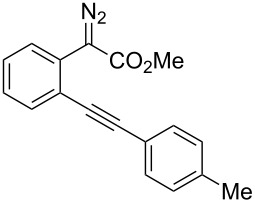 **1d**	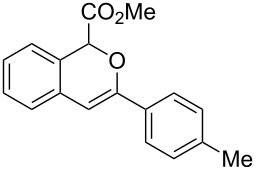 **2d**, 82%	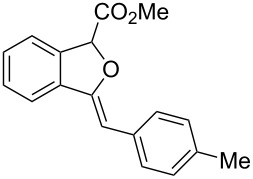 **3d**, <10%
5	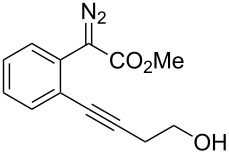 **1e**	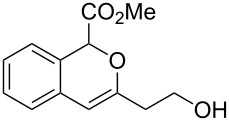 **2e**, 69%	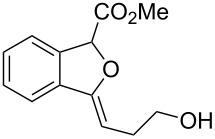 **3e**, Trace
6	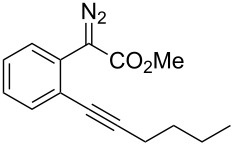 **1f**	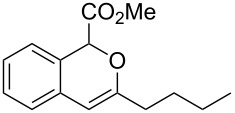 **2f**, 75%	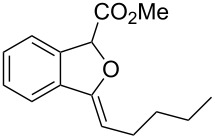 **3f**, Trace
7^c^	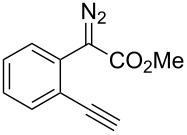 **1g**	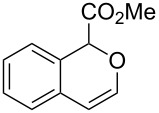 **2g**, 0%	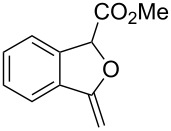 **3g**, 0%

^a^All the reactions were carried out using 0.5 mmol phenyldiazoacetate **1** with 3 mol % of (IPr)AuCl catalyst in 2 mL solvent for 24 h. ^b^Isolated yield. ^c^Only the water insertion product methyl 2-(2-ethynylphenyl)-2-hydroxyacetate (**4g**) was isolated as the major product in a yield of 81%.

**Scheme 2:**
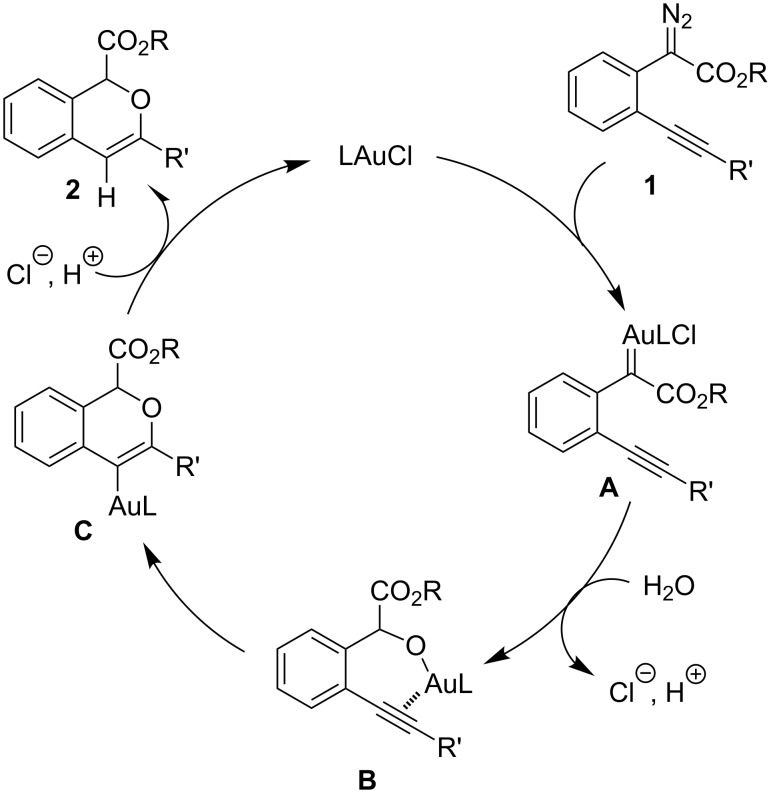
Tentative mechanism.

A tentative mechanism for this gold(I)-catalyzed cascade insertion/cyclization is proposed in Scheme 2. Decomposition of diazo compound **1** by (IPr)AuCl generates gold carbene species **A**, which inserts into the O–H bond of H_2_O to form the chelating intermediate **B**. Subsequently, 6-*endo-dig* attack of the Au(I)-activated triple bond affords the vinylgold intermediate **C**, which is protonated to give the final product **2** with regeneration of the catalyst. This mechanism is supported by the fact that when **4a** was subjected to the gold(I)-catalyzed reaction under identical conditions **2a** and **3a** were obtained in similar yields.

## Conclusion

In summary, we have developed a cascade insertion/cyclization of water with *o-*acetylenyl-substituted phenyldiazoacetates catalyzed by a single Au(I) catalyst. This tandem process provides a novel and straightforward method to synthesize isochromene derivatives. Isochromene and its derivatives frequently occur as structural units in natural products and exhibit interesting biological activities such as antibiotic properties [39–51]. Moreover, this study further demonstrates the possibility to incorporate gold-catalyzed reaction of diazo compounds with various other gold-catalyzed transformations. Further studies to broaden the scope of these reactions are currently underway.

## Experimental

**General**. For chromatography, 200–300 mesh silica gel (Qingdao, China) was employed. ^1^H NMR and ^13^C NMR spectra were recorded on Varian 300 or Bruker ARX 400 spectrometer in CDCl_3_ solution and chemical shifts are reported in parts per million (δ) relative to internal standard TMS (0 ppm). Mass spectra were obtained on a VG ZAB-HS mass spectrometer. Diazo compounds were prepared according to our previous reported procedures. Unless otherwise noted, materials obtained from commercial suppliers were used without further purification.

**General procedure for the gold(I)-catalyzed cascade insertion/cyclization reaction.** Distilled water (0.5 mL) was added to a solution of complex **1** (0.5 mmol) and (IPr)AuCl (3 mol %, 9.3 mg) in DMF (1.5 mL) at room temperature under a N_2_ atmosphere. The reaction mixture was stirred for 24 h at 80 °C. After the mixture cooled to room temperature, it was diluted with ether and water. The aqueous phase was extracted with Et_2_O (2 × 10 mL) and the combined organic extracts were washed with brine (1 × 10 mL), dried over Na_2_SO_4_, and concentrated. Purification by column chromatography on silica gel afforded products **2** and **3**.

**Ethyl 3-phenyl-1*H*-isochromene-1-carboxylate (2a)**. ^1^H NMR (400 MHz, CDCl_3_) δ 7.81 (d, *J =* 6.8 Hz, 2H), 7.41–7.35 (m, 3H), 7.28–7.20 (m, 3H), 7.09 (d, *J =* 7.6 Hz, 1H), 6.36 (s, 1H), 5.83 (s, 1H), 4.15 (m, 2H), 1.19 (t, *J =* 7.2 Hz, 3H); ^13^C NMR (100 MHz, CDCl_3_) δ 170.0, 153.0, 134.2, 130.6, 129.18, 129.16, 128.4, 126.7, 126.2, 126.0, 125.8, 124.1, 100.5, 76.6, 61.7, 14.2; MS (70 eV) *m*/*z* (%): 280 (29) [M^+^], 207 (100), 178 (47), 152 (8); HRMS–ESI (*m*/*z*): [M + H]^+^ calcd for C_18_H_17_O_3_, 281.1172; found, 281.1168.

**Ethyl 3-benzylidene-1,3-dihydroisobenzofuran-1-carboxylate (3a)**. ^1^H NMR (400 MHz, CDCl_3_) δ 7.81 (d, *J =* 7.2 Hz, 2H), 7.75–7.55 (m, 1H), 7.53–7.51 (m, 1H), 7.41–7.32 (m, 4H), 7.18–7.17 (m, 1H), 6.03 (s, 1H), 6.00 (s, 1H), 4.33–4.23 (m, 2H), 1.31 (t, *J =* 7.2 Hz, 3H); ^13^C NMR (100 MHz, CDCl_3_) δ 168.8, 154.8, 136.8, 135.8, 134.6, 129.4, 129.1, 128.5, 128.4, 125.9, 122.3, 120.1, 97.9, 82.6, 62.0, 14.3; MS (70 eV) *m*/*z* (%): 280 (11) [M^+^], 264 (8), 207 (100), 191 (13), 178 (43); HRMS–ESI (*m*/*z*): [M + H]^+^ calcd for C_18_H_17_O_3_, 281.1172; found, 281.1166.

**Methyl 3-(2-bromophenyl)-1*H*-isochromene-1-carboxylate (2b)**. ^1^H NMR (400 MHz, CDCl_3_) δ 7.89–7.86 (m, 1H), 7.64–7.62 (m, 1H), 7.39–7.35 (m, 1H), 7.30–7.19 (m, 4H), 7.11–7.09 (m, 1H), 6.27 (s, 1H), 5.87 (s, 1H), 3.76 (s, 3H); ^13^C NMR (100 MHz, CDCl_3_) δ 170.1, 151.5, 135.5, 133.5, 131.4, 130.1, 129.9, 129.1, 127.3, 127.2, 125.9, 125.5, 124.2, 106.0, 77.2, 52.5; MS (70 eV) *m*/*z* (%): 344 (41, ^79^Br) [M^+^], 285 (100), 206 (36), 178 (96), 151 (17); HRMS–ESI (*m*/*z*): [M + H]^+^ calcd for C_17_H_14_BrO_3_, 345.0120; found, 345.0125.

**Methyl 3-(2-bromobenzylidene)-1,3-dihydroisobenzofuran-1-carboxylate (3b)**. ^1^H NMR (400 MHz, CDCl_3_) δ 8.36–8.34 (m, 1H), 7.66 (d, *J =* 8.0 Hz, 1H), 7.58–7.52 (m, 2H), 7.41–7.39 (m, 2H), 7.34–7.29 (m, 1H), 7.03–7.01 (m, 1H), 6.42 (s, 1H), 6.05 (s, 1H), 3.82 (s, 3H); ^13^C NMR (100 MHz, CDCl_3_) δ 168.9, 155.9, 136.6, 134.9, 134.2, 132.6, 130.0, 129.5, 129.4, 127.3, 127.0, 123.0, 122.1, 96.1, 82.6, 52.8; MS (70 eV) *m*/*z* (%): 344 (13, ^79^Br) [M^+^], 285 (100), 206 (32), 178 (67), 151 (14); HRMS–ESI (*m*/*z*): [M + H]^+^ calcd for C_17_H_14_BrO_3_, 345.0120; found, 345.0127.

**Methyl 3-(3-bromophenyl)-1*H*-isochromene-1-carboxylate (2c)**. ^1^H NMR (400 MHz, CDCl_3_) δ 7.96–7.95 (m, 1H), 7.72–7.71 (m, 1H), 7.49–7.47 (m, 1H), 7.32–7.25 (m, 4H), 7.11–7.09 (m, 1H), 6.37 (s, 1H), 5.86 (s, 1H), 3.71 (s, 3H); ^13^C NMR (100 MHz, CDCl_3_) δ 170.1, 151.3, 136.1, 131.8, 129.8, 129.1, 128.6, 127.1, 126.2, 125.7, 124.2, 124.1, 122.6, 101.4, 76.2, 52.6; MS (70 eV) *m*/*z* (%): 344 (34, ^79^Br) [M^+^], 285 (100), 206 (35), 178 (82), 151 (14); HRMS–ESI (*m*/*z*): [M + H]^+^ calcd for C_17_H_14_BrO_3_, 345.0120; found, 345.0117.

**Methyl 3-(3-bromobenzylidene)-1,3-dihydroisobenzofuran-1-carboxylate (3c)**. ^1^H NMR (400 MHz, CDCl_3_) δ 7.94–7.93 (m, 1H), 7.68–7.66 (m, 1H), 7.55–7.52 (m, 2H), 7.45–7.38 (m, 2H), 7.30–7.27 (m, 1H), 7.22–7.17 (m, 1H), 6.07 (s, 1H), 5.92 (s, 1H), 3.82 (s, 3H); ^13^C NMR (100 MHz, CDCl_3_) δ 168.9, 155.7, 137.8, 136.8, 134.0, 130.8, 129.8, 129.47, 129.44, 128.5, 126.7, 122.5, 122.2, 120.1, 96.4, 82.6, 52.8; MS (70 eV) *m*/*z* (%): 344 (28, ^79^Br) [M^+^], 285 (100), 206 (34), 178 (50), 151 (19); HRMS–ESI (*m*/*z*): [M + H]^+^ calcd for C_17_H_14_BrO_3_, 345.0120; found, 345.0126.

**Methyl 3-*p*-tolyl-1*H*-isochromene-1-carboxylate (2d)**. ^1^H NMR (400 MHz, CDCl_3_) δ 7.69 (d, *J =* 8.4 Hz, 2H), 7.25–7.17 (m, 5H), 7.05 (d, *J =* 6.8 Hz, 1H), 6.31 (s, 1H), 5.83 (s, 1H), 3.67 (s, 3H), 2.36 (s, 3H); ^13^C NMR (100 MHz, CDCl_3_) δ 170.4, 153.1, 139.2, 131.4, 130.7, 129.17, 129.15, 126.5, 126.2, 125.76, 125.71, 123.9, 99.7, 76.5, 52.5, 21.4; MS (70 eV) *m*/*z* (%): 280 (17) [M^+^], 221 (100), 207 (7), 178 (27); HRMS–ESI (*m*/*z*): [M + H]^+^ calcd for C_18_H_17_O_3_, 287.1172; found, 281.1168.

**Methyl 3-(2-hydroxyethyl)-1*H*-isochromene-1-carboxylate (2e)**. ^1^H NMR (400 MHz, CDCl_3_) δ 7.26–7.15 (m, 3H), 6.92 (d, *J =* 7.2 Hz, 1H), 5.74 (s, 1H), 5.70 (s, 1H), 4.09–4.07 (m, 1H), 3.78–3.64 (m, 2H), 3.69 (s, 3H), 2.51–2.47 (m, 2H); ^13^C NMR (100 MHz, CDCl_3_) δ 171.4, 154.0, 130.1, 129.3, 126.5, 126.4, 124.4, 123.2, 102.9, 76.1, 59.7, 52.9, 37.5; MS (70 eV) *m*/*z* (%): 234 (2) [M^+^], 192 (8), 175 (100), 145 (28), 133 (77); HRMS–ESI (*m*/*z*): [M + H]^+^ calcd for C_13_H_15_O_4_, 235.0964; found, 235.0960.

**Methyl 3-butyl-1*H*-isochromene-1-carboxylate (2f). **^1^H NMR (400 MHz, CDCl_3_) δ 7.21–7.11 (m, 3H), 6.89 (d, *J =* 7.6 Hz, 1H), 5.67 (s, 1H), 5.58 (s, 1H), 3.68 (s, 3H), 2.77 (t , *J =* 7.6 Hz, 2H), 1.65–1.58 (m , 2H), 1.42–1.36 (m , 2H), 0.94 (t , *J =* 7.2 Hz, 2H); ^13^C NMR (100 MHz, CDCl_3_) δ 170.5, 157.3, 130.7, 129.0, 126.0, 124.7, 123.0, 99.9, 76.3, 52.4, 33.5, 28.6, 22.4, 14.0; MS (70 eV) *m*/*z* (%): 246 (12) [M^+^], 187 (100), 115 (16); HRMS–ESI (*m*/*z*): [M + H]^+^ calcd for C_15_H_19_O_3_, 247.1328; found, 247.1322.

**Methyl 2-(2-ethynylphenyl)-2-hydroxyacetate (4g)**. ^1^H NMR (400 MHz, CDCl_3_) δ 7.53 (d, *J =* 7.2 Hz, 1H), 7.37–7.36 (m, 2H), 7.32–7.29 (m, 1H), 5.63 (d, *J =* 5.6 Hz, 1H), 3.75 (s, 3H), 3.59 (d, *J =* 5.2 Hz, 1H), 3.31 (s , 1H); ^13^C NMR (100 MHz, CDCl_3_) δ 174.0, 140.5, 133.4, 129.4, 128.5, 127.1, 121.5, 82.2, 81.3, 71.4, 53.2; MS (70 eV) *m*/*z* (%): 190 (100) [M^+^], 159 (69), 132 (57), 103 (53); HRMS–ESI (*m*/*z*): [M + H]^+^ calcd for C_11_H_11_O_3_, 191.0702; found, 191.0699.

## Supporting Information

File 1 ^1^H and ^13^C NMR spectra.
