# Supplementary material for: Sequential Au(I)-catalyzed reaction of water with o-acetylenyl-substituted phenyldiazoacetates
Source: Beilstein J Org Chem. 2011 May 18;7:631–7. doi: 10.3762/bjoc.7.74 (PMC3107481; doi:10.3762/bjoc.7.74)
Supplement: File 1 — 1H and 13C NMR spectra. [file Beilstein_J_Org_Chem-07-631-s001.pdf]

Supporting Information

for

Sequential Au(I)-catalyzed reaction of water with *o*-acetylenyl-substituted phenyldiazoacetates

Lei Zhou, Yizhou Liu, Yan Zhang and Jianbo Wang*

Address: Beijing National Laboratory of Molecular Sciences (BNLMS), Key Laboratory of Bioorganic Chemistry and Molecular Engineering of Ministry of Education, College of Chemistry, Peking University, Beijing 100871, China

Email: Jianbo Wang - wangjb@pku.edu.cn

*Corresponding author

^{1}H and ^{13}C NMR spectra

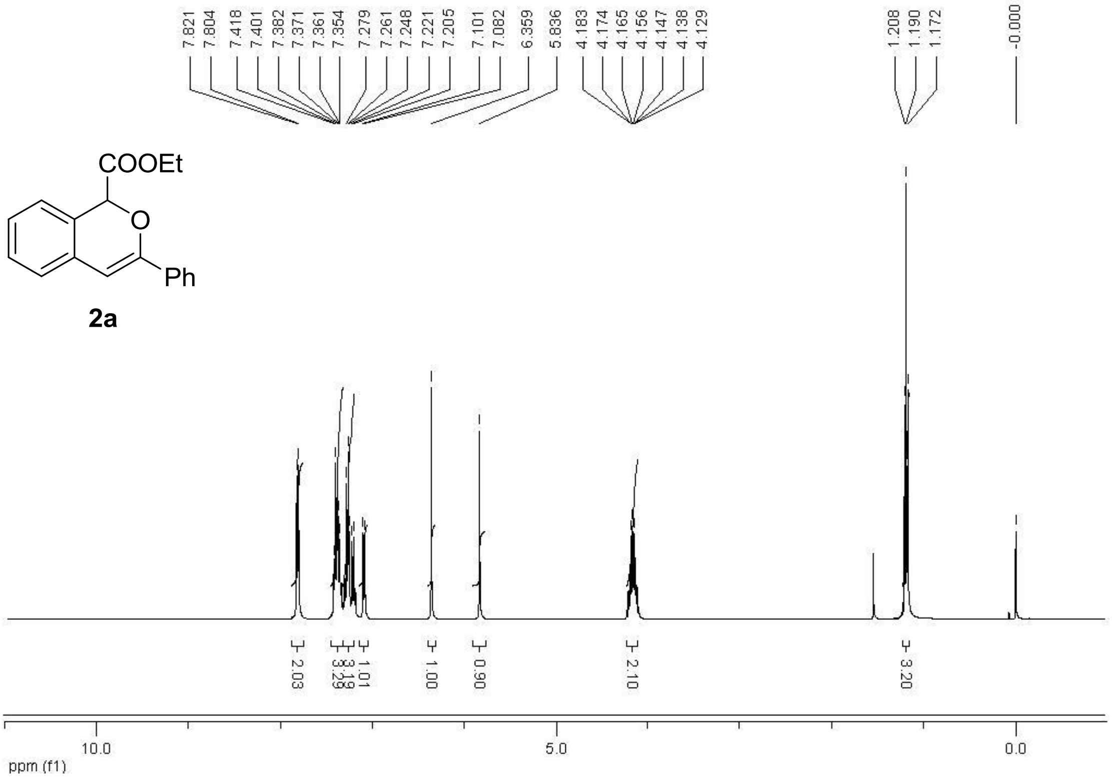
7.821
7.804
7.418
7.401
7.382
7.371
7.361
7.354
7.279
7.261
7.248
7.221
7.205
7.101
7.082
6.359
5.836
4.183
4.174
4.165
4.156
4.147
4.138
4.129
1.208
1.190
1.172
-0.000
COOEt
O
Ph
2a
2.03
3.29
3.19
1.01
1.00
0.90
2.10
3.20
10.0
5.0
0.0
ppm (f1)

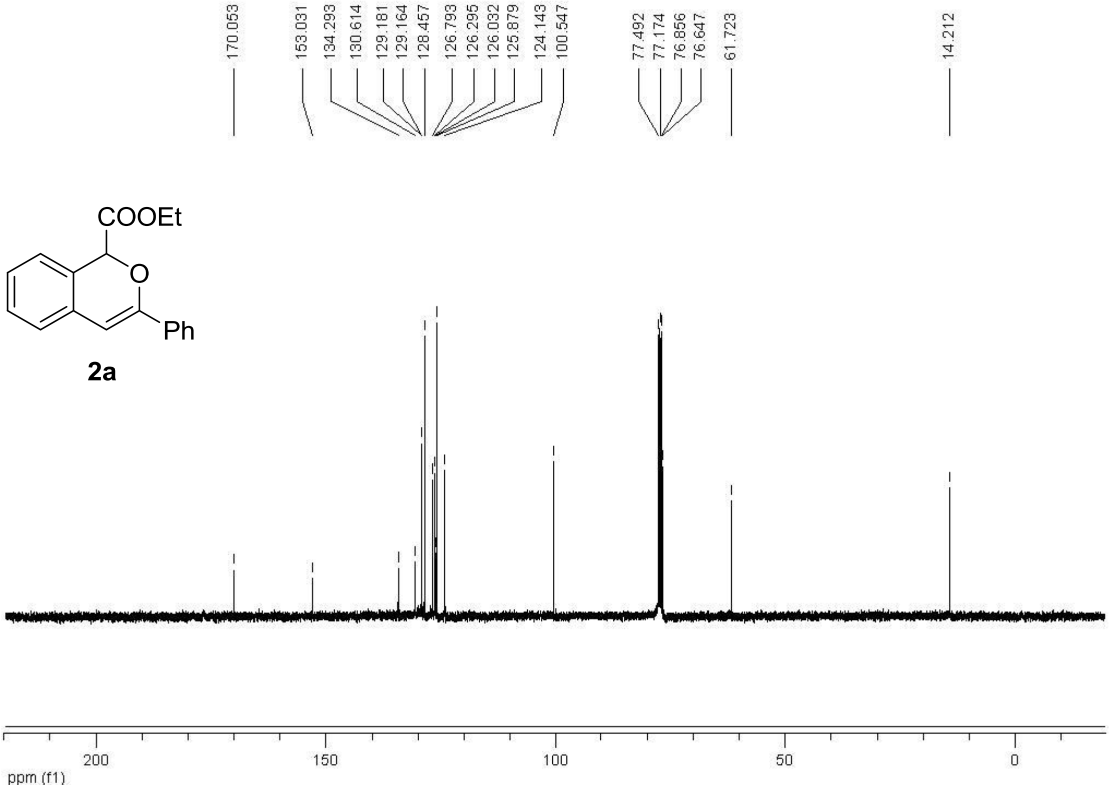
170.053
153.031
134.293
130.614
129.181
129.164
128.457
126.793
126.295
126.032
125.879
124.143
100.547
77.492
77.174
76.856
76.647
61.723
14.212
COOEt
O
Ph
2a
200
150
100
50
0
ppm (f1)

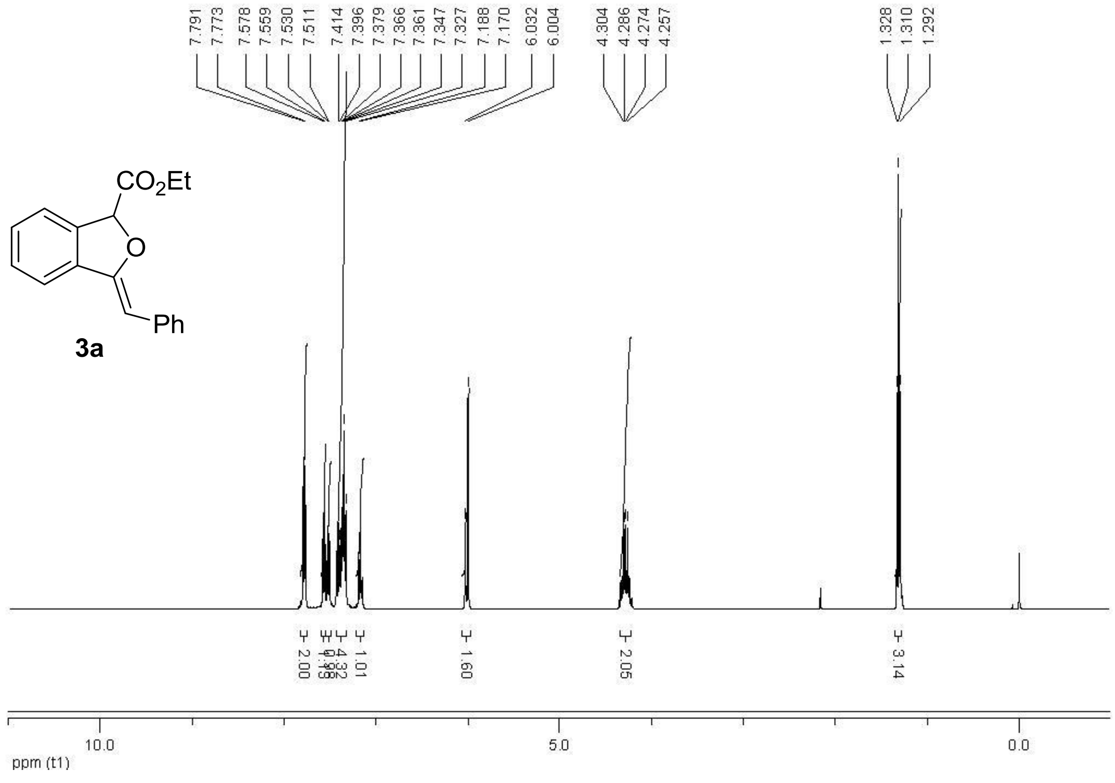
7.791 7.773 7.578 7.559 7.530 7.511 7.414 7.396 7.379 7.366 7.361 7.347 7.327 7.188 7.170 6.032 6.004
4.304 4.286 4.274 4.257
1.328 1.310 1.292
CO2Et
O
Ph
3a
2.00
4.32
1.01
1.60
2.05
3.14
10.0
5.0
0.0
ppm (t1)

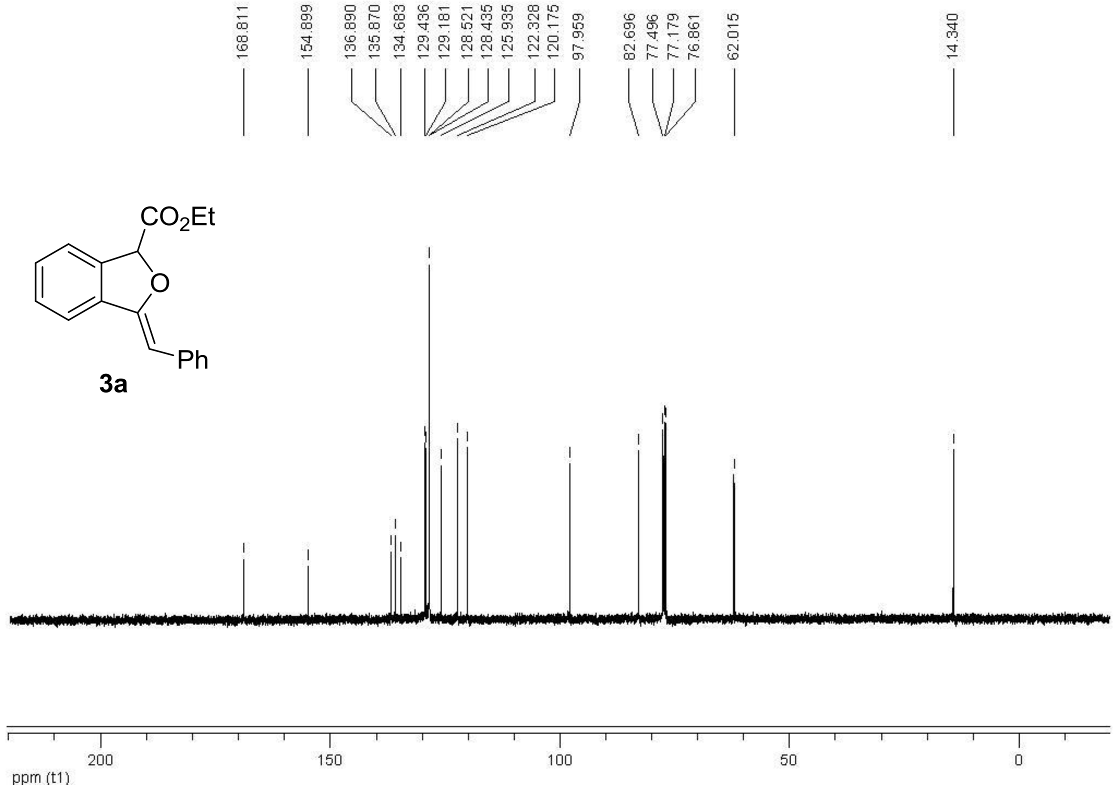
168.811
154.899
136.890 135.870 134.683 129.436 129.181 128.521 128.435 125.935 122.328 120.175
97.959
82.696 77.496 77.179 76.861
62.015
14.340
CO2Et
O
Ph
3a
200
150
100
50
0
ppm (t1)

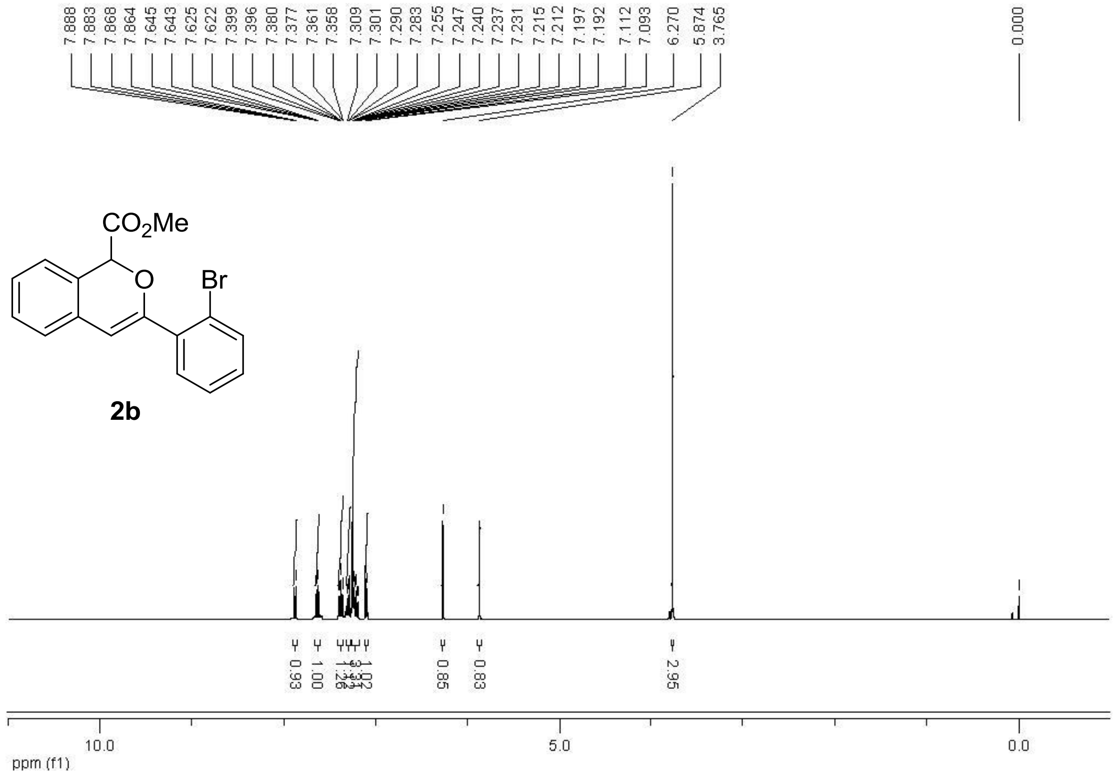
7.888 7.883 7.868 7.864 7.645 7.643 7.625 7.622 7.399 7.396 7.380 7.377 7.361 7.358 7.309 7.301 7.290 7.283 7.255 7.247 7.240 7.237 7.231 7.215 7.212 7.197 7.192 7.112 7.093 6.270 5.874 3.765
0.000
CO2Me
O
Br
2b
0.93
1.00
1.02
0.85
0.83
2.95
10.0
5.0
0.0
ppm (f1)

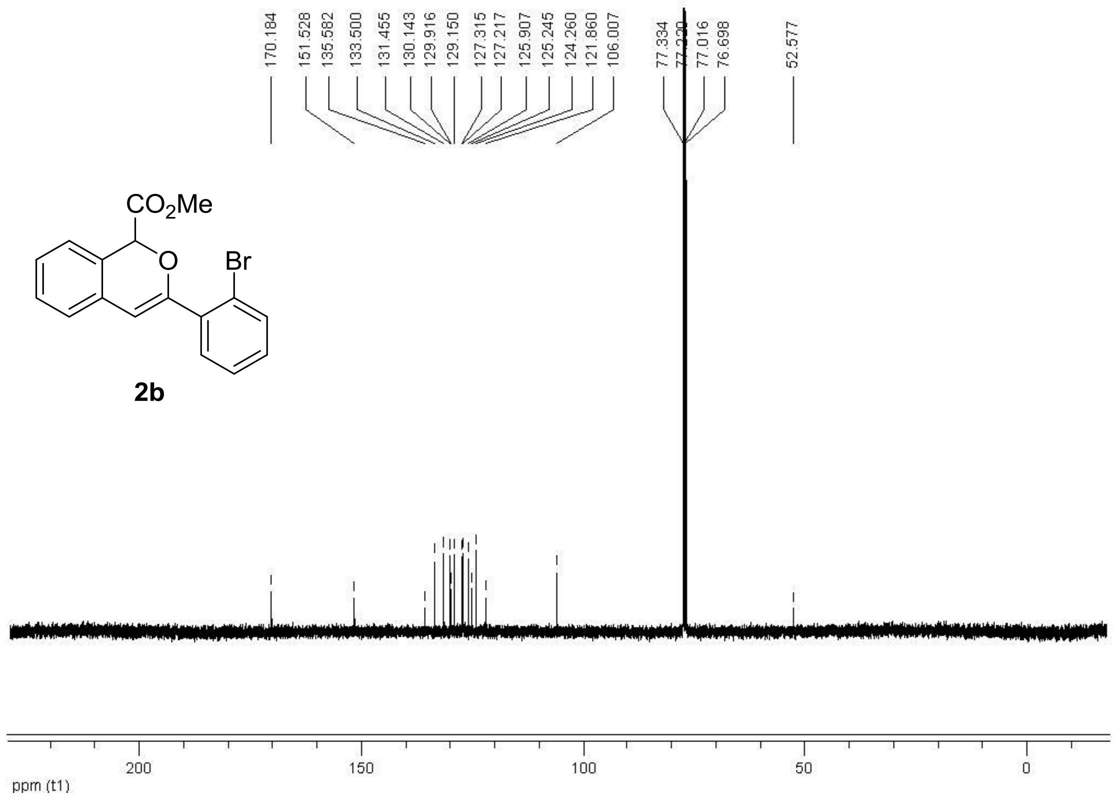
170.184 151.528 135.582 133.500 131.455 130.143 129.916 129.150 127.315 127.217 125.907 125.245 124.260 121.860 106.007
77.334 77.016 76.698
52.577
CO2Me
O
Br
2b
200
150
100
50
0
ppm (t1)

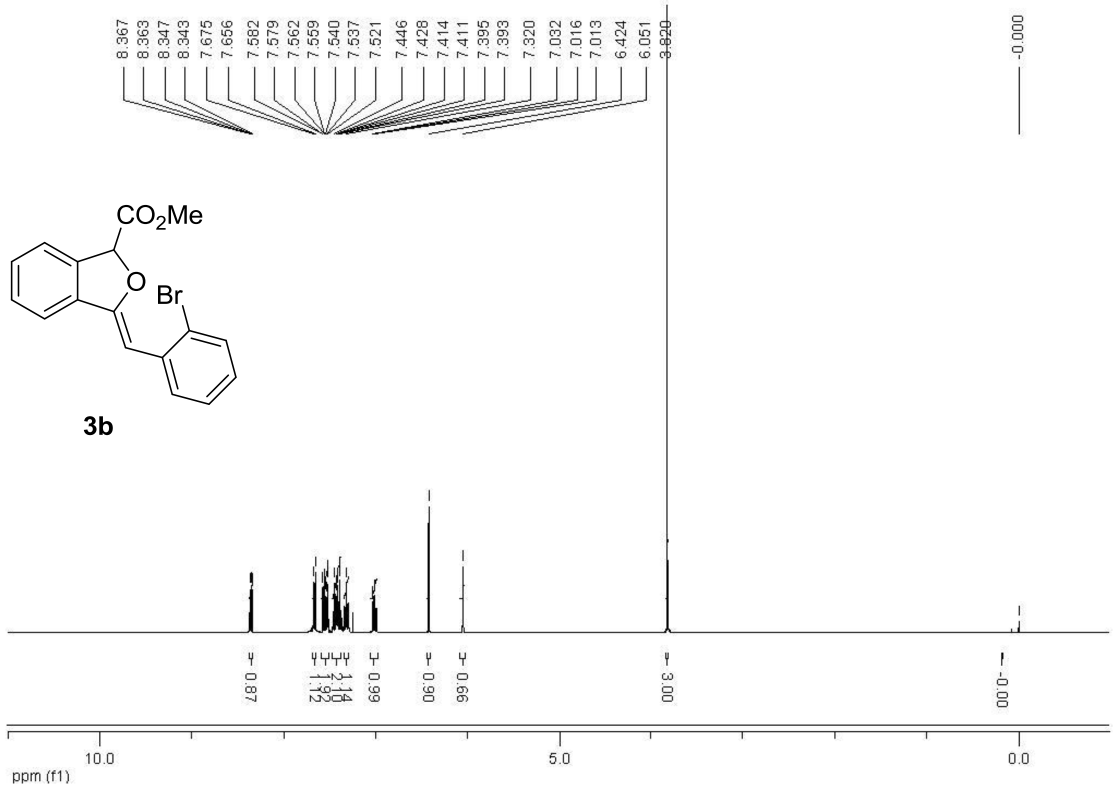
8.367
8.363
8.347
8.343
7.675
7.656
7.582
7.579
7.562
7.559
7.540
7.537
7.521
7.446
7.428
7.414
7.411
7.395
7.393
7.320
7.032
7.016
7.013
6.424
6.051
3.820
-0.000
CO_2Me
O
Br
3b
0.87
1.92
1.72
2.10
1.14
0.99
0.90
0.66
3.00
-0.00
10.0
5.0
0.0
ppm (f1)

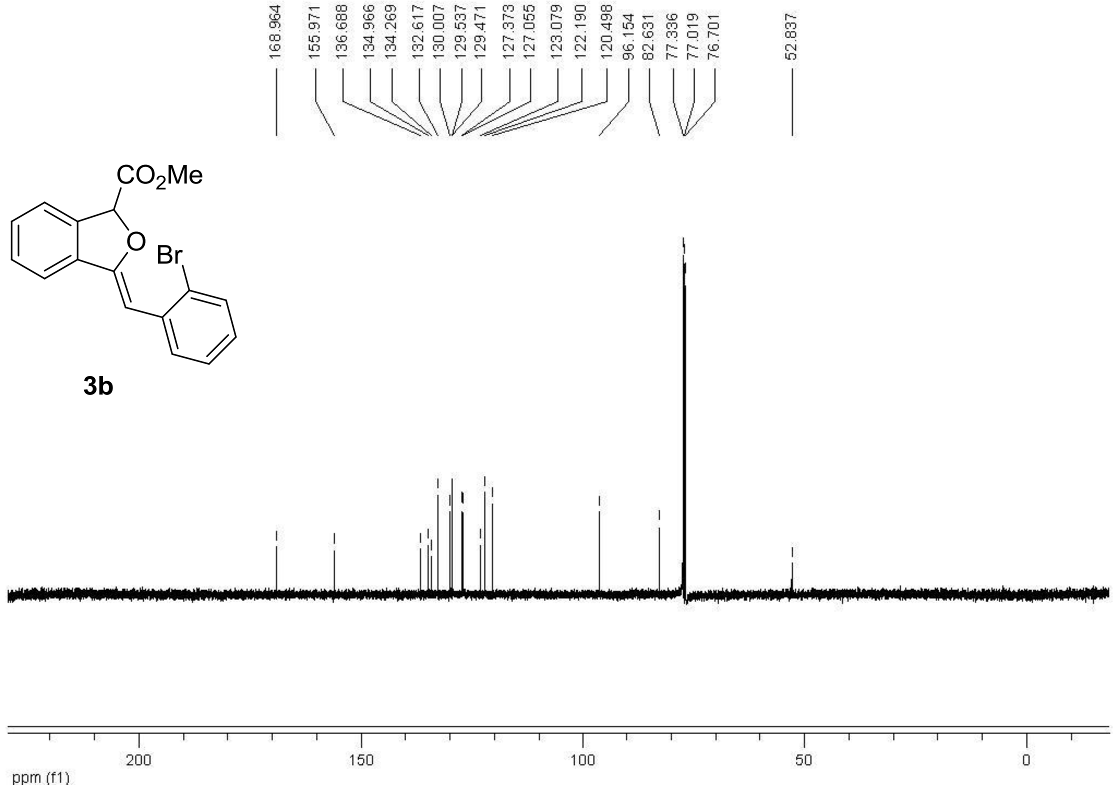
168.964
155.971
136.688
134.966
134.269
132.617
130.007
129.537
129.471
127.373
127.055
123.079
122.190
120.498
96.154
82.631
77.336
77.019
76.701
52.837
CO_2Me
O
Br
3b
200
150
100
50
0
ppm (f1)

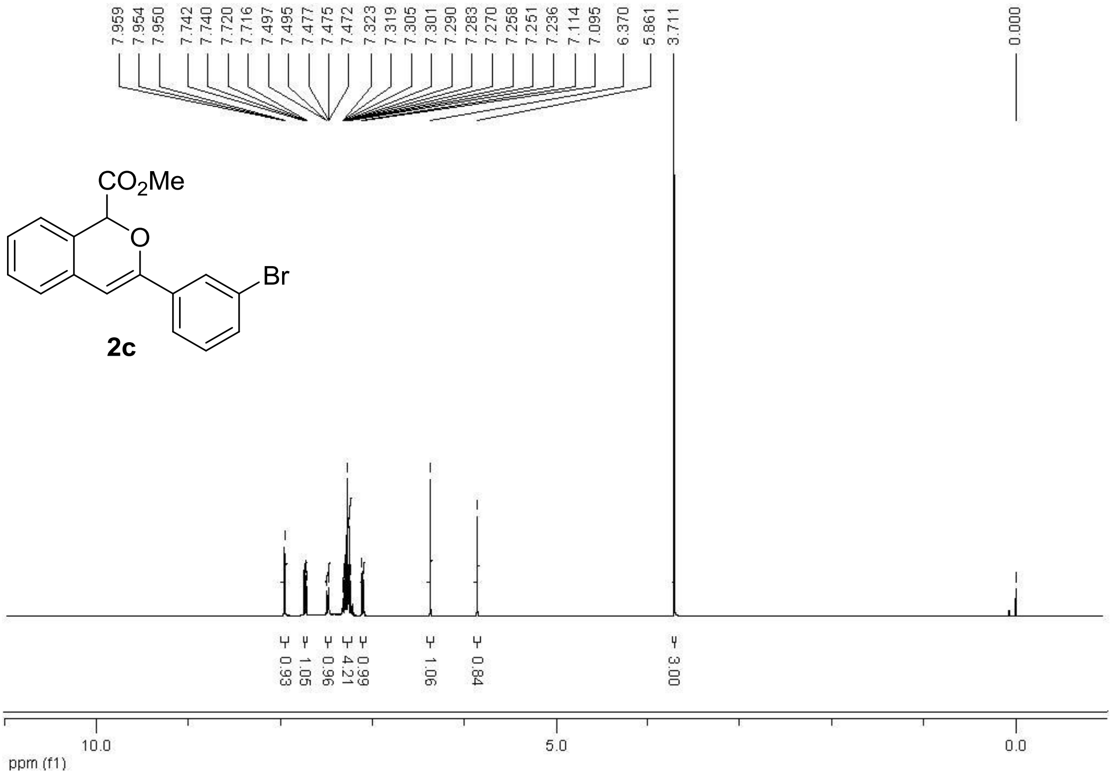
7.959
7.954
7.950
7.742
7.740
7.720
7.716
7.497
7.495
7.477
7.475
7.472
7.323
7.319
7.305
7.301
7.290
7.283
7.270
7.258
7.251
7.236
7.114
7.095
6.370
5.861
3.711
0.000
CO2Me
O
Br
2c
0.93
1.05
0.96
4.21
0.99
1.06
0.84
3.00
10.0
5.0
0.0
ppm (f1)

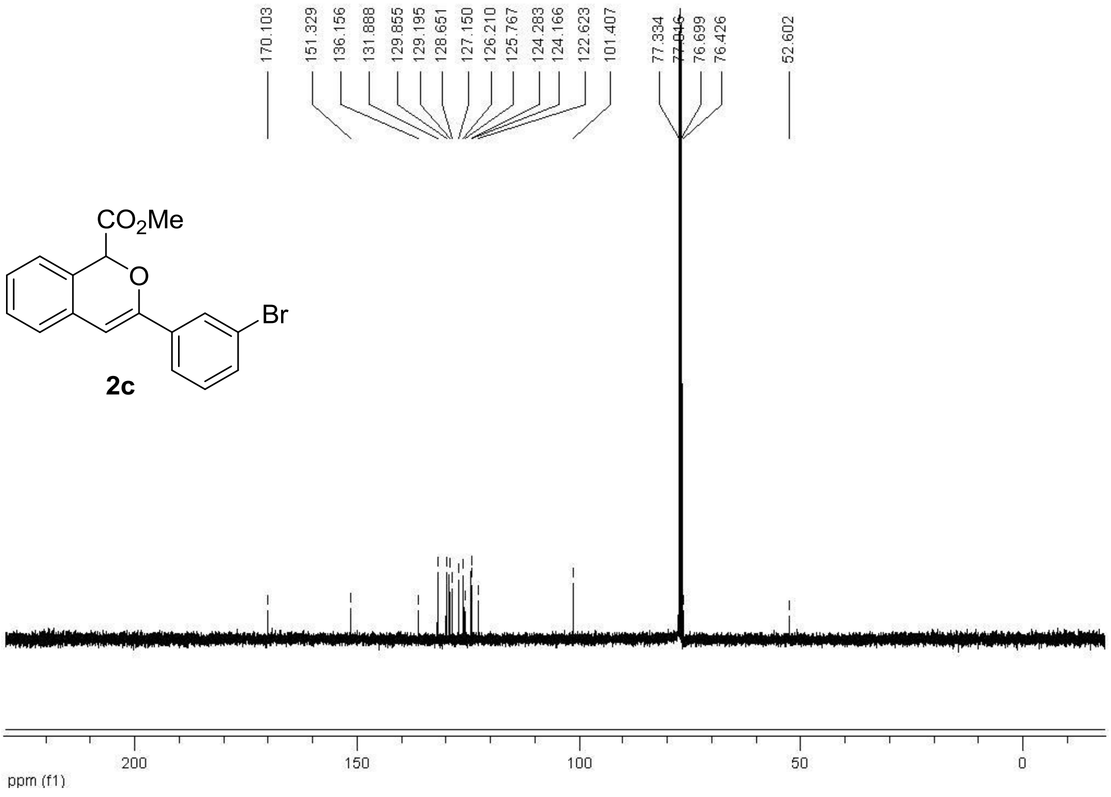
170.103
151.329
136.156
131.888
129.855
129.195
128.651
127.150
126.210
125.767
124.283
124.166
122.623
101.407
77.334
77.016
76.699
76.426
52.602
CO2Me
O
Br
2c
200
150
100
50
0
ppm (f1)

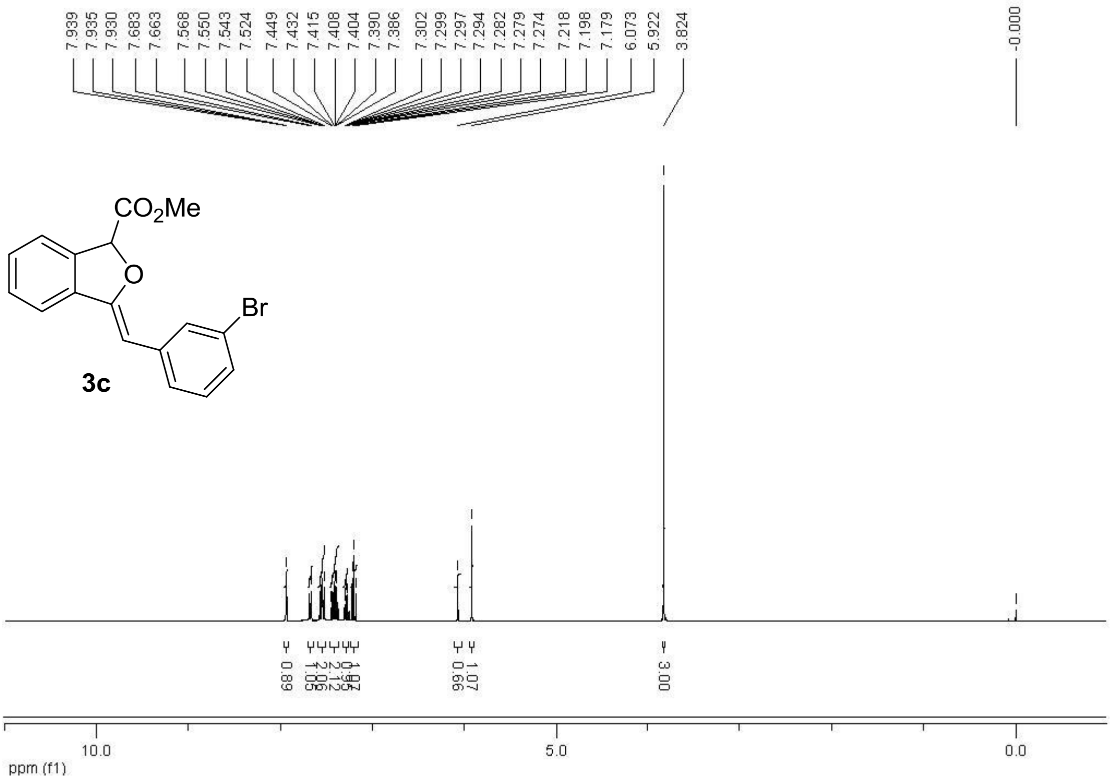
7.939
7.935
7.930
7.683
7.663
7.568
7.550
7.543
7.524
7.449
7.432
7.415
7.408
7.404
7.390
7.386
7.302
7.299
7.297
7.294
7.282
7.279
7.274
7.218
7.198
7.179
6.073
5.922
3.824
-0.000
CO2Me
O
Br
3c
0.89
0.66
1.07
3.00
10.0
5.0
0.0
ppm (f1)

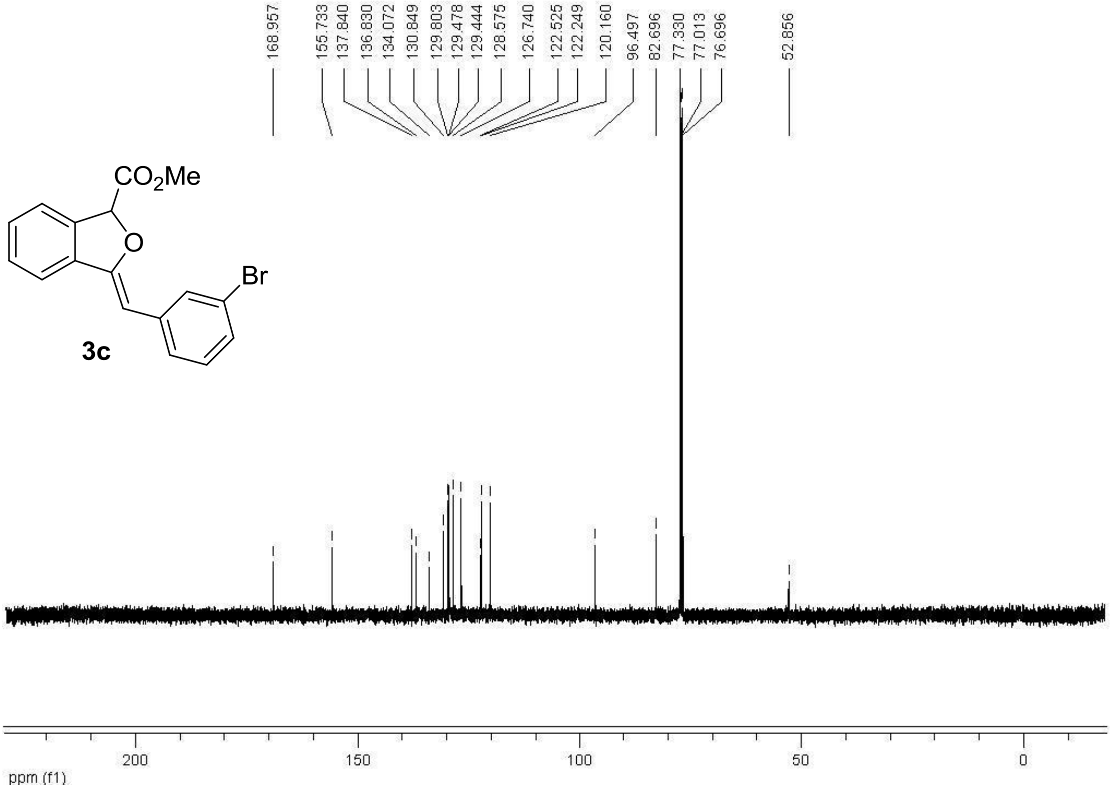
168.957
155.733
137.840
136.830
134.072
130.849
129.803
129.478
129.444
128.575
126.740
122.525
122.249
120.160
96.497
82.696
77.330
77.013
76.696
52.856
CO2Me
O
Br
3c
200
150
100
50
0
ppm (f1)

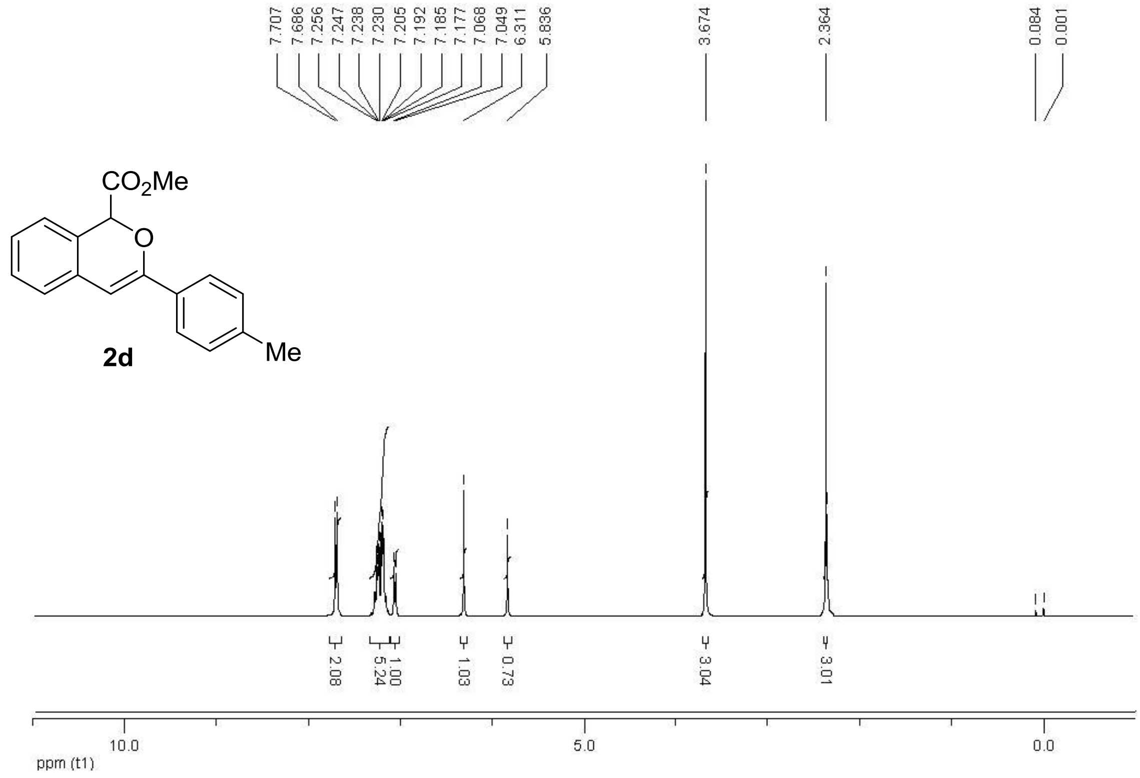
7.707
7.686
7.256
7.247
7.238
7.230
7.205
7.192
7.185
7.177
7.068
7.049
6.311
5.836
3.674
2.364
0.084
0.001
CO_2Me
O
Me
2d
2.08
5.24
1.00
1.03
0.73
3.04
3.01
10.0
5.0
0.0
ppm (t1)

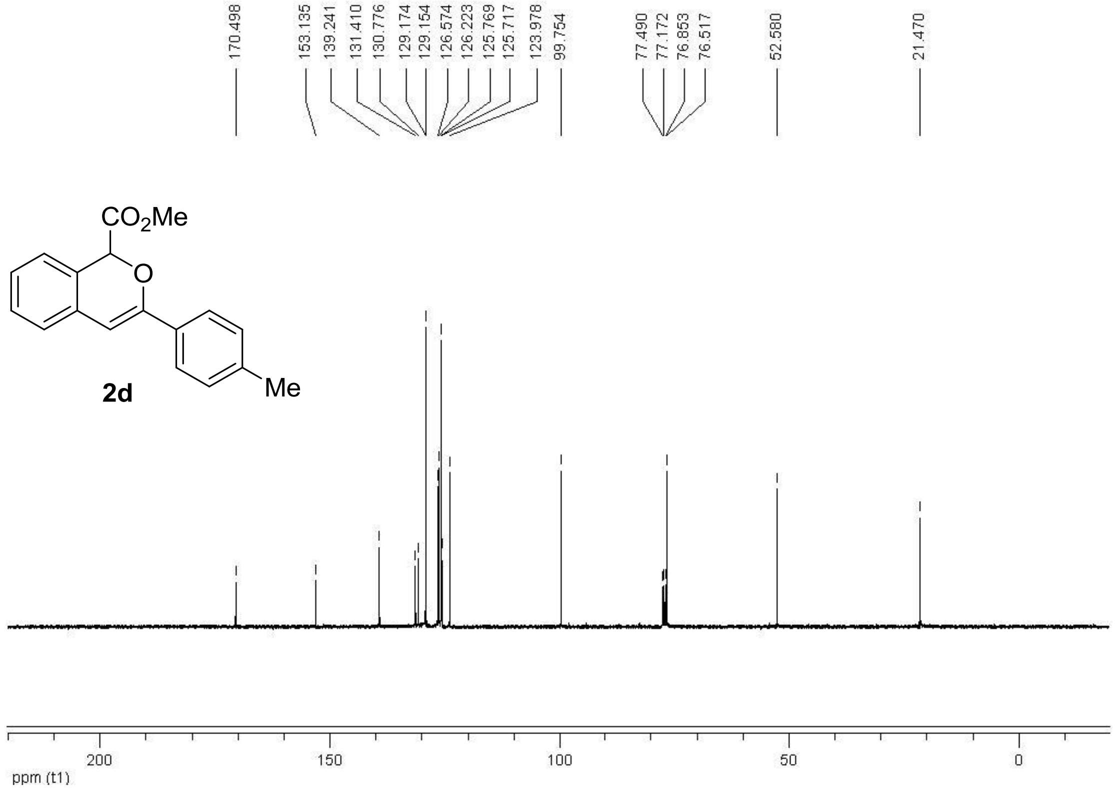
170.498
153.135
139.241
131.410
130.776
129.174
129.154
126.574
126.223
125.769
125.717
123.978
99.754
77.490
77.172
76.853
76.517
52.580
21.470
CO_2Me
O
Me
2d
200
150
100
50
0
ppm (t1)

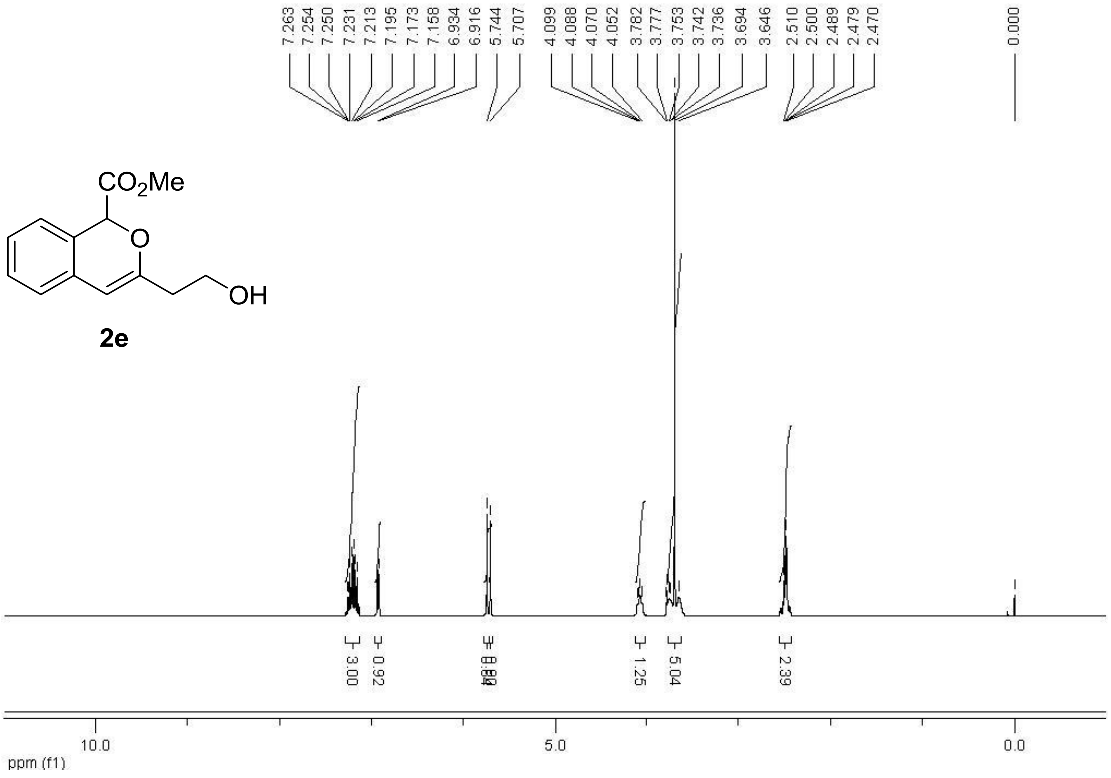
7.263 7.254 7.250 7.231 7.213 7.195 7.173 7.158 6.934 6.916 5.744 5.707 4.099 4.088 4.070 4.052 3.782 3.777 3.753 3.742 3.736 3.694 3.646 2.510 2.500 2.489 2.479 2.470 0.000
CO2Me
O
OH
2e
3.00 0.92 1.25 5.04 2.39
10.0 5.0 0.0
ppm (f1)

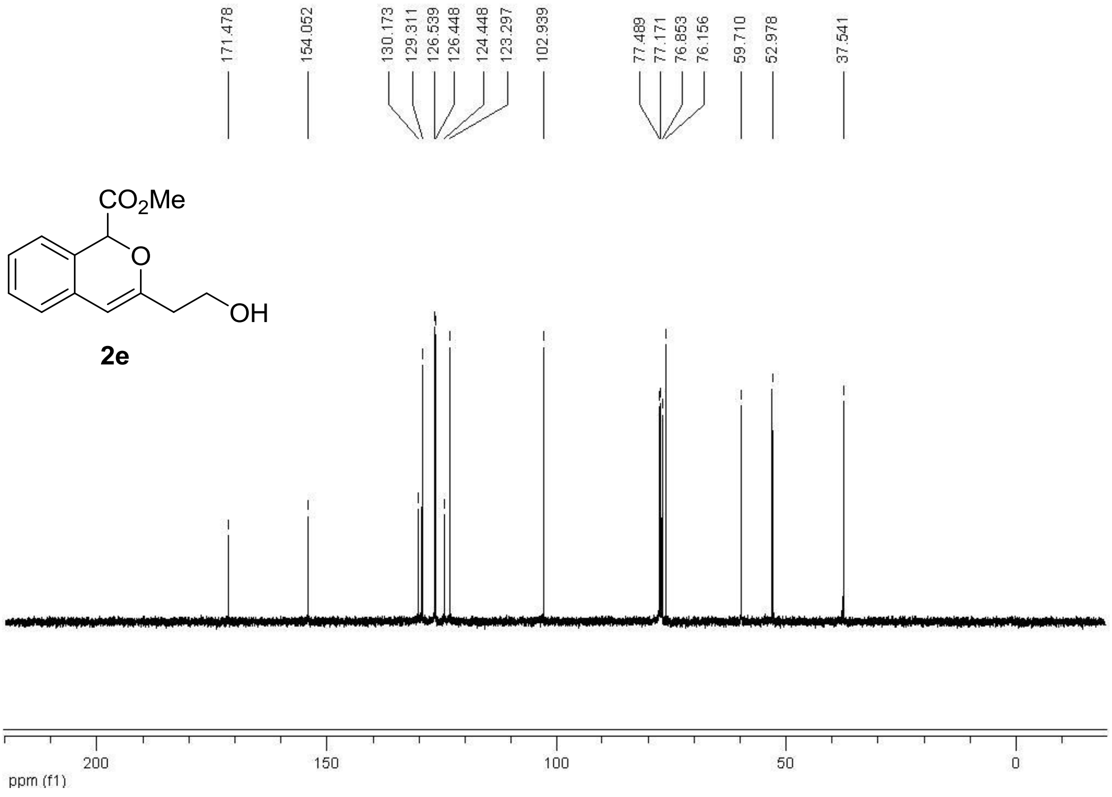
171.478 154.052 130.173 129.311 126.539 126.448 124.448 123.297 102.939 77.489 77.171 76.853 76.156 59.710 52.978 37.541
CO2Me
O
OH
2e
200 150 100 50 0
ppm (f1)

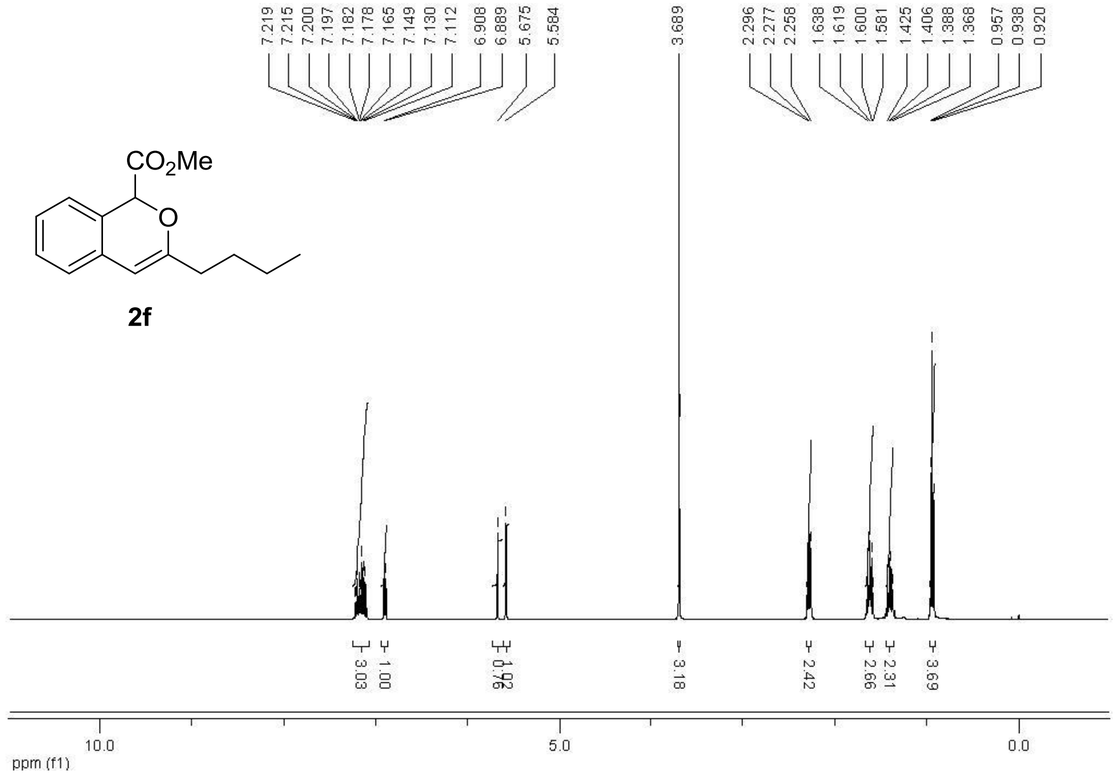
7.219
7.215
7.200
7.197
7.182
7.178
7.165
7.149
7.130
7.112
6.908
6.889
5.675
5.584
3.689
2.296
2.277
2.258
1.638
1.619
1.600
1.581
1.425
1.406
1.388
1.368
0.957
0.938
0.920
CO2Me
O
2f
3.03
1.00
3.18
2.42
2.66
2.31
3.69
10.0
5.0
0.0
ppm (f1)

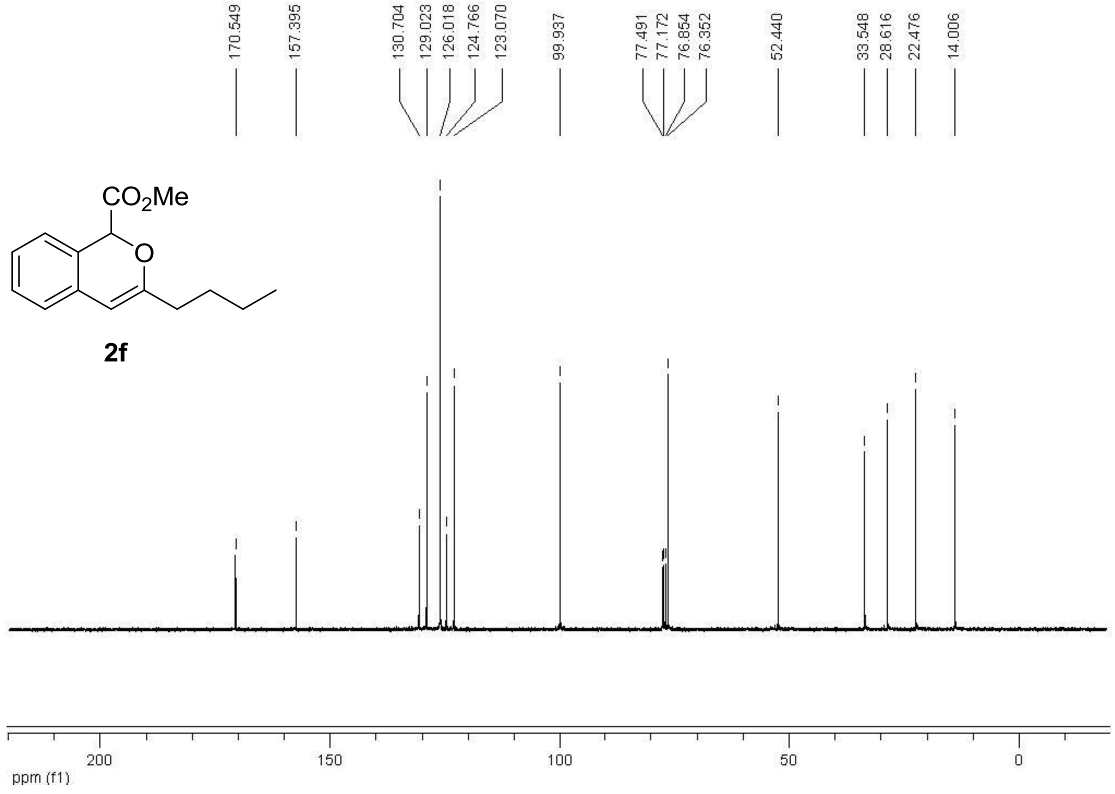
170.549
157.395
130.704
129.023
126.018
124.766
123.070
99.937
77.491
77.172
76.854
76.352
52.440
33.548
28.616
22.476
14.006
CO2Me
O
2f
200
150
100
50
0
ppm (f1)

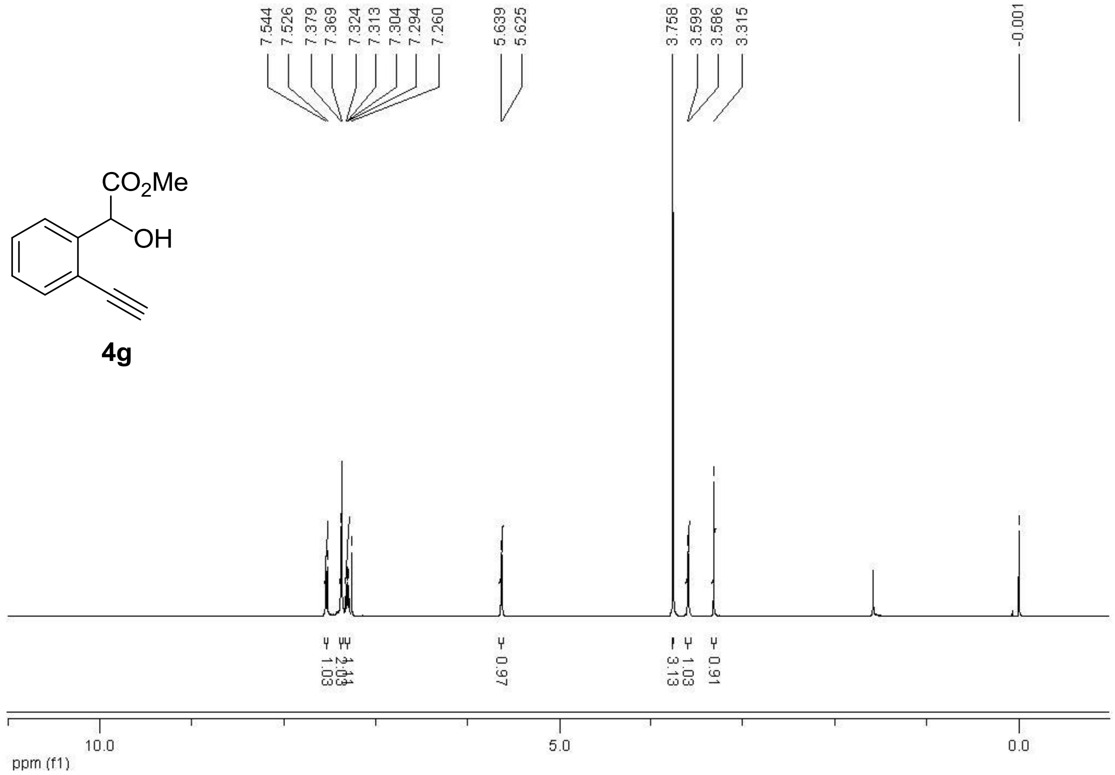
7.544
7.526
7.379
7.369
7.324
7.313
7.304
7.294
7.260
5.639
5.625
3.758
3.599
3.586
3.315
-0.001
CO2Me
OH
4g
1.03
1.11
2.03
0.97
3.13
1.03
0.91
10.0
5.0
0.0
ppm (f1)

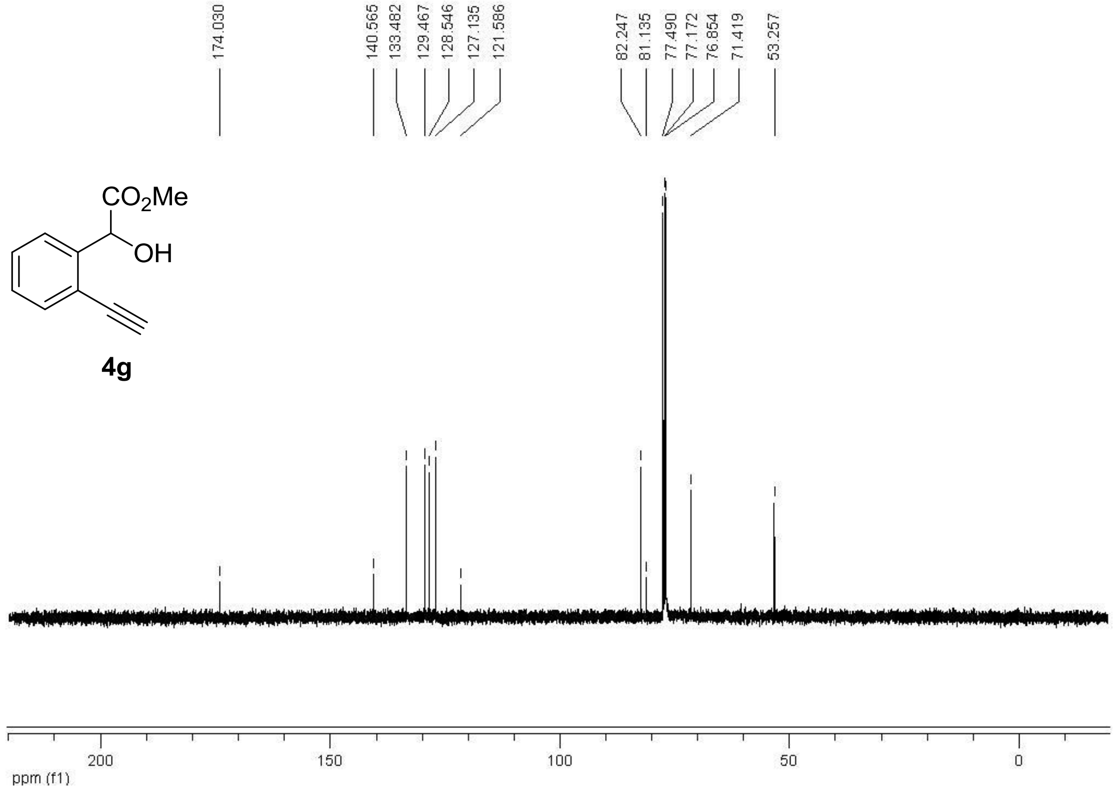
174.030
140.565
133.482
129.467
128.546
127.135
121.586
82.247
81.135
77.490
77.172
76.854
71.419
53.257
CO2Me
OH
4g
200
150
100
50
0
ppm (f1)
